# The Epidemiology of Hip Fracture among Subjects with Pyogenic Liver Abscess (PLA): A Nationwide Population-Based Study

**DOI:** 10.1155/2020/5901962

**Published:** 2020-02-12

**Authors:** Chieh-Cheng Hsu, Jih-Yang Ko, Cheng-Li Lin, Horng-Chaung Hsu, Hsien-Te Chen, Che-Chen Lin, Shu-Jui Kuo

**Affiliations:** ^1^Department of Orthopedic Surgery, Kaohsiung Chang Gung Memorial Hospital and Chang Gung University College of Medicine, Kaohsiung, Taiwan; ^2^Center for Shockwave Medicine and Tissue Engineering, Kaohsiung Chang Gung Memorial Hospital, Kaohsiung, Taiwan; ^3^Management Office for Health Data, China Medical University Hospital, Taichung, Taiwan; ^4^School of Medicine, China Medical University, Taichung, Taiwan; ^5^Department of Orthopedic Surgery, China Medical University Hospital, Taichung, Taiwan; ^6^Department of Sports Medicine, College of Health Care, China Medical University, Taichung, Taiwan; ^7^Spine Center, China Medical University Hospital, China Medical University, Taichung, Taiwan

## Abstract

Pyogenic liver abscess (PLA) is a potentially fatal disease that can stimulate prominent systemic inflammation. Osteoporotic hip fracture is a major complication of systemic inflammation. This study tried to determine the epidemiology of hip fractures among PLA patients. All subjects admitted due to PLA during 1999∼2010 were assessed, excluding the subjects with a history of high energy trauma, malignancy, and previous hip fracture. We matched the control subjects to PLA patients according to age, gender, and the coding of osteoporosis by 1 : 4 ratio. The PLA patients had a 1.17-fold risk of hip fracture than the controls (aHR = 1.17, 95% CI = 1.07–1.29) after adjusting for gender, age, and comorbidities. Considering death as the competing event of suicide, the PLA patients had 1.10-fold suicide risk (aHR = 1.10, 95% CI: 1.00–1.21) than the control subjects under the competing risks regression model. The cumulative incidence of hip fracture was higher in the PLA cohort (log-rank test, *p* < 0.001). When compared to the controls, the fracture risk was 18.4-fold (aHR = 18.4, 95% CI = 13.0–26.1) for the PLA patients admitted 2-3 times per year and 46.0-fold (aHR = 46.0, 95% CI = 31.2–67.8) for the PLA patients admitted ≧4 times per year. The impact of PLA is more prominent among the subjects aged <45 years (aHR = 2.81, 95% CI = 1.42–5.56). Preventive measures for hip fracture might be warranted for PLA patients.

## 1. Introduction

Pyogenic liver abscess (PLA) is a potentially lethal disease with the mortality rate of up to 19% [1]. PLA can be seen in the subjects with diabetes, existing hepatobiliary disease, and in individuals who have undergone invasive procedures [2]. In recent years, the incidence of PLA has surged persistently all over the world. In the United States, the incidence of PLA has grown from 2.7 per 100,000 individuals in 1994 to 4.1 per 100,000 individuals in 2005 [3]. In Taiwan, the prevalence was higher than that in the United States, increasing from 10.83 per 100,000 individuals in 2000 to 15.45 per 100,000 individuals in 2011 [4]. However, the long-term sequelae among the survivors of PLA are not completely understood at present. The increasing population of PLA, as well as the PLA survivors, underscores the importance of identifying the long-term sequelae of PLA. The use of oral corticosteroids is significantly associated with an increased risk of developing PLA with a dose-dependent effect [5, 6].

Osteoporosis is the major sequelae under systemic inflammation, which can be explained by the perspective of altered energy metabolism, evolution, and immunologic and neuroendocrine factors [7]. Osteoporotic hip fractures account for one-fourth of geriatric fractures necessitating hospitalization with the mortality rate reaching 20% [8].

Osteoporotic fractures are prevalent among individuals with hepatobiliary diseases, including primary biliary cirrhosis, chronic hepatitis C infection, or primary sclerosing cholangitis [9]. Recently, cholangitis has been recognized to be associated with a higher risk of hip fracture [8]. Despite the surging prevalence of PLA and the considerable mortality rate after hip fracture, the epidemiologic information of hip fracture among PLA patients is not determined at present. In our study, we want to investigate the pertinent epidemiology of hip fracture among the individuals with PLA history.

## 2. Materials and Methods

### 2.1. Data Source

The National Health Insurance (NHI) program was established by the Taiwanese government in 1995. It is single-payer based and nearly 99.9% Taiwanese citizens were enrolled. The database, named National Health Insurance Research Database (NHIRD), was released by the National Health Research Institute (NHRI) and contained all health claim data from Taiwan NHI. The history of disease diagnosis was obtained from the inpatient files under the coding system of the International Classification of Disease, Ninth Revision, Clinical Modification (ICD-9-CM). To protect the privacy of the subjects, the initial identification number of each individual was removed and replaced by the scrambled random number. Our study was approved by the Research Ethics Committee of China Medical University (CMUH-104-REC2-115-R3).

### 2.2. Study Population

This population-based cohort study tried to investigate the epidemiology of hip fracture among PLA patients. All of the subjects admitted with the de novo coding of PLA (ICD-9-CM code: 572.0) between January 1999 and December 2010 were all recruited for assessment. The date when the diagnosis of PLA was initially coded was defined as the index date. The control subjects without PLA coding were matched to the PLA patients according to gender, age (every 5 years), and the coding of osteoporosis (ICD-9-CM 733.0 and 733.1) by 1 : 4 ratio and recruited at the index date. The individuals with high energy trauma (presence of E coding) and cancer (ICD-9-CM 140-208) at the index date and the follow-up period and with the history of a previous hip fracture (ICD-9-CM 820) at the index date were excluded for both groups [8]. All of the subjects were followed until the withdrawal from the insurance, the occurrence of hip fracture, or until December 31, 2011.

The comorbidities analyzed in our study included liver cirrhosis, coronary artery disease (CAD), diabetes (DM), epilepsy, hypertension, stroke, and end-stage renal disease.

### 2.3. Statistical Analysis

We used the two-sample *t*-test for the comparison of continuous variables and the chi-square test for categorical variables. The Kaplan–Meier method was utilized to plot the survival curves for the two cohorts with the log-rank test chosen to express the significance of difference. The hazard ratios (HRs), adjusted hazard ratios (aHR), and 95% confidence intervals (CIs) were presented by using crude and adjusted Cox proportional hazard models to compare the risk of hip fracture in both cohorts.

The proportional hazard model assumption was also examined using a test of scaled Schoenfeld residuals. In the model evaluating the hip fracture risk throughout the overall follow-up period, the results of the test revealed a significant relationship between Schoenfeld residuals for PLA and follow-up time, suggesting that the proportionality assumption was conformed (*p* value = 0.06). The subhazard ratio (SHR) was also calculated after further controlling for the competing risk of deaths using the Fine and Gray method as a sensitivity analysis. All statistical analyses were performed using SAS statistical software, version 9.4 (SAS Institute Inc., Cary, NC). The figure of the cumulative incidence curve was plotted by R software. The significant criteria were set below 0.05 for the *p* value of the two-sided testing [9].

## 3. Results

There were 25530 patients in the PLA cohort and 102120 subjects in the control cohort in our study (Table 1). In the study population, 63.0% were male. The mean age of the PLA cohort and the control cohort was 59.4 ± 14.6 and 59.3 ± 14.6 years, respectively. The composition of gender, age, and osteoporosis was homogenous between the two groups. The prevalence of liver cirrhosis, CAD, diabetes, epilepsy, hypertension, stroke, ESRD, and COPD was significantly higher in the PLA cohort (all *p* < 0.001).

**Figure 1:**
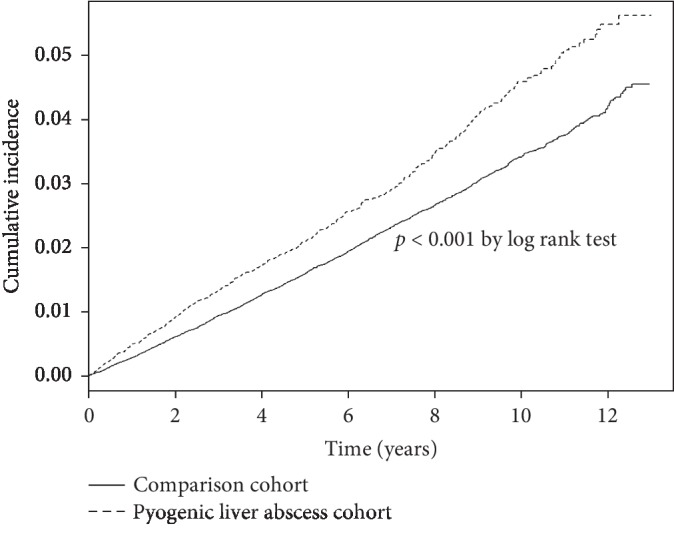
The Kaplan–Meier survival curves for PLA and comparison cohorts. The incidence of hip fracture with substantially higher in the PLA cohort (*p* < 0.001 by log-rank test).

The incidence of hip fracture was 44.8 per 10000 person-years in the PLA cohort and 33.7 per 10000 person-years in the control cohort (Table 2). The patients with PLA had a 1.17-fold fracture risk than the subjects without PLA (aHR = 1.17, 95% CI = 1.07–1.29) after adjusting for gender, age, and all comorbidities. The cumulative incidence of hip fracture was significantly higher in the PLA cohort (log-rank test *p* < 0.01) (Figure 1). Considering death as the competing event of suicide, the PLA patients had 1.10-fold suicide risk (aHR = 1.10, 95% CI: 1.00–1.21) than the control subjects under the competing risks regression model (Table 3). The risk of hip fracture was significantly higher among subjects from 45 to 64 years of age (aHR = 3.48, 95% CI = 2.53–4.78) and ≧65 years old (aHR = 21.4, 95% CI = 15.7–29.1). The male subjects had lower risk of hip fracture (aHR = 0.65, 95% CI = 0.60–0.70) than the female subjects. The patients with osteoporosis (aHR = 1.57, 95% CI = 1.32–1.85), liver cirrhosis (aHR = 1.80, 95% CI = 1.37–2.37), DM (aHR = 1.38, 95% CI = 1.26–1.50), hypertension (aHR = 1.23, 95% CI = 1.11–1.36), stroke (aHR = 1.64, 95% CI = 1.46–1.85), ESRD (aHR = 2.05, 95% CI = 1.52–2.75), and COPD (aHR = 1.71, 95% CI = 1.48–1.97) had higher risk of hip fracture (Table 2).  Table 4 demonstrated the incidence and adjusted hazard ratio of hip fracture stratified by the admission frequency under the coding of PLA. The risk of hip fracture among the subjects who were admitted 2-3 times per year was 18.4-fold (aHR = 18.4, 95% CI = 13.0–26.1) compared to the control cohort. The risk of hip fracture increased to 46.0-fold (aHR = 46.0, 95% CI = 31.2–67.8) for the PLA patients who were admitted ≧4 times per year. The higher the admission frequency under the coding of PLA, the higher the risk of hip fracture (*p* for trend <0.001).

Table 5 compared the incidence and adjusted hazard ratio of hip fracture between the PLA and non-PLA cohorts stratified by gender, age, and comorbidities. PLA is associated with higher risk of hip fracture in both males (aHR = 1.25, 95% CI = 1.09–1.44) and females (aHR = 1.18, 95% CI = 1.04–1.34). The impact of PLA on the occurrence of hip fracture is more prominent among the subjects aged <45 years old (aHR = 2.81, 95% CI = 1.42–5.56).

## 4. Discussion

In our study, we showed that the subjects with the coding of PLA had a 1.17-fold risk of suffering from hip fracture than the individuals without PLA after adjusting for age, gender, and pertinent comorbidities. We also showed that the admission frequency is positively correlated with hip fracture risk. We demonstrated that the impact of PLA on the occurrence of hip fracture is more prominent among the individuals aged <45 years old and among the subjects with pertinent comorbidities (aHR = 1.15, 95% CI = 1.03∼1.28). Considering death as the competing event of suicide, the PLA patients had 1.10-fold suicide risk (aHR = 1.10, 95% CI: 1.00–1.21) than the control subjects under the competing risks regression model.

This study is the first one demonstrating the epidemiology of hip fracture among PLA patients.

The “three-pillar theory” tried to describe the possible mechanisms of inflammatory bone loss, including disturbed energy metabolism, evolution, and immunologic and neuroendocrine factors [7]. The receptor activator of nuclear factor-*κ*B (RANK) is widely known as an immunologic factor and osteoclastic activator [7, 8]. The mortality rate of osteoporotic hip fracture is as high as 20% within 1 year. We thus tried to determine the epidemiology of hip fracture in the disease entities that are capable of stimulating systemic inflammation, such as PLA. Previous studies showed that >90% PLA patients were presented with fever and >25% with positive bacterial culture, highlighting the phenomena that PLA can trigger substantial systemic inflammation [1]. It is worth-investigating whether the extent of PLA-induced inflammation could lead to osteoporotic hip fracture.

Hsu et al. have shown that the risk of hip fracture was substantially higher among the patients afflicted with cholangitis than that among the control subjects (aHR = 1.29, 95% CI = 1.03–1.61) in one population-based study [8]. Angulo et al. also showed that osteoporosis could be observed in 15% individuals afflicted with primary sclerosing cholangitis, and the incidence rate was 23.8-fold than the gender and age-matched controls [10]. These results suggested the correlation between hepatobiliary inflammation and bone loss. In our study, we showed that the patients with PLA had 1.17 times the possibility of suffering from hip fractures than the matched control individuals. We also demonstrated that the possibility of hip fracture among the subjects who were admitted 2-3 times per year under the coding of PLA was 18.4-fold compared to the control cohort, and the risk increased to 46.0-fold among the PLA patients who were admitted ≧4 times per year. These findings suggested that the higher the severity of PLA, the higher the risk of hip fracture.

Age, female gender, osteoporosis, liver cirrhosis, DM, hypertension, stroke, ESRD, and COPD were also correlated with a higher risk of hip fracture in our model. These findings inferred from our model were in keeping with the previous studies, consolidating the validity of our model [8, 11].

It is intriguing that the impact of PLA on the occurrence of hip fracture is more prominent among the subjects aged <45 years old (aHR = 2.81, 95% CI = 1.42–5.56). We hypothesize that the subjects <45 years old have fewer established risk factors, such as age, ESRD, and stroke, for hip fractures, so the impact of PLA is less diluted [12, 13]. The correlation between PLA, age, and hip fracture warrants further investigation.

There are limitations to our study. First, not all of the risk factors for hip fractures could be retrieved from the NHIRD, such as body height, weight, parent hip fracture history, and the consumption of tobacco and alcohol. Second, there were substantial hip fracture patients who were coded with ICD-9-CM 820.8, the coding including transcervical and pertrochanteric fractures, in the NHIRD. As a result, we cannot differentiate the transcervical and pertrochanteric fractures among a substantial portion of hip fracture patients.

## 5. Conclusions

We showed that PLA is associated with a higher risk for hip fracture than the gender, age, and the status of osteoporosis-matched control subjects. The admission frequency is positively correlated with the risk of hip fracture. The impact of PLA is more prominent among the subjects aged <45 years old and the presence of comorbidities. Preventive measures for hip fracture, such as antiosteoporotic regimen, might be considered for PLA patients.

## Figures and Tables

**Table 1:** Comparison of baseline demographic profiles and history of comorbidities between the control and PLA cohorts.

Variable	Control cohort *N* = 102120 (%)	PLA cohort *N* = 25530 (%)	*p* value
Age, year (SD)	59.3 (14.6)	59.4 (14.6)	0.29
Sex			>0.99
Female	37752 (37.0)	9438 (37.0)	
Male	64368 (63.0)	16092 (63.0)	
Comorbidities			
Osteoporosis	2164 (2.1)	541 (2.1)	>0.99
Liver cirrhosis	706 (0.7)	743 (2.9)	<0.01
CAD	6138 (6.0)	2554 (10.0)	<0.01
DM	14748 (14.4)	10264 (40.2)	<0.01
Epilepsy	304 (0.3)	131 (0.5)	<0.01
Hypertension	12459 (12.2)	7421 (29.1)	<0.01
Stroke	5477 (5.4)	2084 (8.2)	<0.01
ESRD	532 (0.5)	594 (2.3)	<0.01
COPD	3056 (3.0)	1293 (5.1)	<0.01

PLA: pyogenic liver abscess; CAD: coronary artery disease; DM: diabetes mellitus; ESRD: end-stage renal disease; COPD: chronic obstructive pulmonary disease.

**Table 2:** Incidence and adjusted hazard ratio of hip fracture stratified by PLA, age, gender, and comorbidities.

Variables	Event	PYs	Rate	Crude HR 95% CI	Adjusted HR 95% CI
PLA					
No	2067	613796	33.7	Ref	Ref
Yes	616	137586	44.8	1.34 (1.22–1.46)	1.17 (1.07–1.29)
Age group, years					
<45	42	141361	2.97	Ref	Ref
45–64	446	364211	12.3	4.16 (3.03–5.70)	3.48 (2.53–4.78)
≧65	2195	245811	89.3	31.1 (22.9–42.3)	21.4 (15.7–29.1)
Sex					
Female	1479	277483	53.3	Ref	Ref
Male	1204	473900	25.4	0.48 (0.44–0.51)	0.65 (0.60–0.70)
Osteoporosis					
No	2518	739214	34.1	Ref	Ref
Yes	165	12169	136	4.05 (3.46–4.75)	1.57 (1.32–1.85)
Liver cirrhosis					
No	2630	746050	35.3	Ref	Ref
Yes	53	5332	99.4	2.89 (2.20–3.80)	1.8 (1.37–2.37)
CAD					
No	2316	712661	32.5	Ref	Ref
Yes	367	38721	94.8	2.99 (2.67–3.34)	1.08 (0.96–1.22)
DM					
No	1846	621865	29.7	Ref	Ref
Yes	837	129518	64.6	2.21 (2.03–2.39)	1.38 (1.26–1.50)
Epilepsy					
No	2665	749657	35.6	Ref	Ref
Yes	18	1726	104	3.00 (1.89–4.77)	1.52 (0.95–2.43)
Hypertension					
No	1866	662023	28.2	Ref	Ref
Yes	817	89360	91.4	3.36 (3.09–3.65)	1.23 (1.11–1.36)
Stroke					
No	2291	720446	31.8	Ref	Ref
Yes	392	30936	127	4.13 (3.71–4.60)	1.64 (1.46–1.85)
ESRD					
No	2637	747524	35.3	Ref	Ref
Yes	46	3858	119	3.48 (2.60–4.66)	2.05 (1.52–2.75)
COPD					
No	2445	734019	33.3	Ref	Ref
Yes	238	17364	137	4.23 (3.70–4.83)	1.71 (1.48–1.97)

Model adjusted for gender, age, and comorbidities. PLA: pyogenic liver abscess; PYs: person-years; rate: incidence rate per 10000 person-years; HR: hazard ratio; CI: confidence interval; CAD: coronary artery disease; DM: diabetes mellitus; ESRD: end-stage renal disease; COPD: chronic obstructive pulmonary disease.

**Table 3:** The subhazard ratio of hip fracture estimated through the competing risks regression model.

Variable	Pyogenic liver abscess		*p* value
	No	Yes	
Crude SHR (95% CI)	1 (reference)	1.24 (1.13, 1.36)	<0.001
Adjusted SHR^†^ (95% CI)	1 (reference)	1.10 (1.00, 1.21)	0.04

SHR: subhazard ratio, crude SHR: relative subhazard ratio; adjusted SHR^†^: multivariable analysis including all factors in the univariable Cox model. ^*∗*^*p* < 0.05; ^*∗∗*^*p* < 0.01; ^*∗∗∗*^*p* < 0.001.

**Table 4:** Incidence of hip fracture stratified by the frequency of admission under the coding of PLA.

	*N*	Event	PYs	Rate	Adjusted HR 95% CI
Comparison cohort	102120	2067	613796	33.7	Ref
PLA cohort					
1	23869	544	137051	39.7	1.08 (0.98–1.19)
2-3	564	35	378	927	18.4 (13.0–26.1)
≥4	1097	37	157	2353	46.0 (31.2–67.8)
*p* for trend					<0.001

Model adjusted for gender, age, and comorbidities. PLA: pyogenic liver abscess; *N*: number of subjects within the cohort; event: hip fracture numbers; PYs: person-years; rate: incidence rate per 10000 person-years; HR: hazard ratio.

**Table 5:** Incidence and hazard ratio of hip fracture among the PLA and control cohorts stratified by gender and age.

Variables	PLA						Crude HR (95% CI)	Adjusted HR (95% CI)
	No (*N* = 102120)			Yes (*N* = 25530)				
	Event	Person-years	Rate^#^	Event	Person-years	Rate^#^		
Gender								
Female	1147	227197	50.5	332	50286	66.0	1.32 (1.17, 1.49)	1.18 (1.04, 1.34)
Male	920	386600	23.8	284	87300	32.5	1.37 (1.20, 1.57)	1.25 (1.09, 1.44)
Age, years								
<45	22	114174	1.93	20	27187	7.36	3.84 (2.09, 7.03)	2.81 (1.42, 5.56)
45–64	310	296756	10.5	136	67456	20.2	1.97 (1.61, 2.41)	1.23 (0.98, 1.53)
≧65	1735	202867	85.5	460	42944	107.1	1.27 (1.14, 1.40)	1.10 (0.99, 1.23)

PLA: pyogenic liver abscess; HR = hazard ratio, CI = confidence interval. PYs: person-years; rate^#^: incidence rate per 10000 person-years. Model adjusted for gender, age, and comorbidities.

## Data Availability

The data used to support the findings of this study are available from the corresponding author (e-mail: b90401073@gmail.com) upon request.

## Abbreviations

PLA: Pyogenic liver abscess
NHIRD: National Health Insurance Research Database
NHRI: National Health Research Institute
ICD-9-CM: International Classification of Disease, Ninth Revision, Clinical Modification
HRs: Hazard ratios
aHRs: Adjusted hazard ratios
CIs: Confidence intervals
RANK: Receptor activator of nuclear factor-*κ*B.
