# Corrigendum to “The Epidemiology of Hip Fracture among Subjects with Pyogenic Liver Abscess (PLA): A Nationwide Population-Based Study”

**DOI:** 10.1155/2020/8401548

**Published:** 2020-06-22

**Authors:** Chieh-Cheng Hsu, Jih-Yang Ko, Cheng-Li Lin, Horng-Chaung Hsu, Hsien-Te Chen, Che-Chen Lin, Shu-Jui Kuo

**Affiliations:** ^1^Department of Orthopedic Surgery, Kaohsiung Chang Gung Memorial Hospital and Chang Gung University College of Medicine, Kaohsiung, Taiwan; ^2^Center for Shockwave Medicine and Tissue Engineering, Kaohsiung Chang Gung Memorial Hospital, Kaohsiung, Taiwan; ^3^Management Office for Health Data, China Medical University Hospital, Taichung, Taiwan; ^4^School of Medicine, China Medical University, Taichung, Taiwan; ^5^Department of Orthopedic Surgery, China Medical University Hospital, Taichung, Taiwan; ^6^Department of Sports Medicine, College of Health Care, China Medical University, Taichung, Taiwan; ^7^Spine Center, China Medical University Hospital, China Medical University, Taichung, Taiwan

In the article titled “The Epidemiology of Hip Fracture among Subjects with Pyogenic Liver Abscess (PLA): A Nationwide Population-Based Study” [1], in Section 2.1, “Data Source,” of the manuscript, the IRB number needs to be changed to CMUH104-REC2-115(CR-3). The Section 2.1 is shown below:

## 2.1. Data Source

The National Health Insurance (NHI) program was established by the Taiwanese government in 1995. It is single-payer based and nearly 99.9% Taiwanese citizens were enrolled. The database, named National Health Insurance Research Database (NHIRD), was released by the National Health Research Institute (NHRI) and contained all health claim data from Taiwan NHI. The history of disease diagnosis was obtained from the inpatient files under the coding system of the International Classification of Disease, Ninth Revision, Clinical Modification (ICD-9-CM). To protect the privacy of the subjects, the initial identification number of each individual was removed and replaced by the scrambled random number. Our study was approved by the Research Ethics Committee of China Medical University CMUH104-REC2-115(CR-3).

## References

[1] Hsu C.-C., Ko J.-Y., Lin C.-L. (2020). The Epidemiology of Hip Fracture among Subjects with Pyogenic Liver Abscess (PLA): A Nationwide Population-Based Study. *BioMed Research International*.

